# 

**Published:** 1872-09

**Authors:** 


☞ Should any of our Subscribers or Exchanges fail to get THE COMPANION regularly, they will confer a favor by notifying us of the fact.

☞ Our Mailing Clerk will faithfully forward THE COMPANION. Subscribers will please notify Post-masters that it will reach their offices, if not lost on the way between the 15th and 20th of every month. Should it fail to come, after a reasonable time, notify the Editors, and it will be sent.

☞ We respectfully solicit Original Articles, Reports of Societies, Clinics Home and Foreign Correspondence, News, etc., etc., of interest to medical readers.

☞ To insure publication, articles should be practical, brief as possible, and carefully written, on ONE side of the paper. Articles should be received by the 15th of one month to insure publication in the next. ☞ Friends, send in your articles. ☜

☞ All Subscribers and others will please write their names, post-offices, county and State *plainly.* ☞ *Remember, this is now required by the Post-Master General.*

